# How and when artificial intelligence adoption promotes employee knowledge sharing? The role of paradoxical leadership and technophilia

**DOI:** 10.3389/fpsyg.2025.1573587

**Published:** 2025-05-21

**Authors:** Xiaoyue Hu, Hongyan Gao, Tesfaye Agafari, Minnie Qing Zhang, Rong Cong

**Affiliations:** School of Economics and Management, Beijing Jiaotong University, Beijing, China

**Keywords:** artificial intelligence adoption, paradoxical leadership, technophilia, employee learning opportunities, employee knowledge sharing

## Abstract

**Introduction:**

The integration of artificial intelligence (AI) into workplaces has transformed organizational operations, yet its impact on employee knowledge sharing remains underexplored. While AI adoption enhances learning and collaboration, the extent to which employees engage in knowledge sharing depends on leadership styles and attitudes toward technology. This study investigates how AI adoption promotes knowledge sharing through employee learning opportunities, while considering the moderating roles of paradoxical leadership and technophilia.

**Methods:**

A survey was conducted with 364 employees across various organizations to examine the proposed relationships. Structural equation modeling (SEM) was employed to test the mediation effect of learning opportunities and the moderating effects of paradoxical leadership and technophilia.

**Results:**

The findings reveal that AI adoption positively influences employee knowledge sharing, with learning opportunities serving as a key mediating factor. Furthermore, paradoxical leadership and technophilia amplify this relationship, indicating that employees with a strong affinity for technology and those working under paradoxical leaders are more likely to leverage AI for knowledge sharing.

**Discussion:**

These results provide important implications for organizations seeking to maximize the benefits of AI adoption. Managers should foster a paradoxical leadership style and support employees in developing a positive attitude toward technology to enhance knowledge-sharing behaviors. Future research should explore additional contextual factors influencing AI-driven knowledge sharing.

## 1 Introduction

The rapid advancement of artificial intelligence (AI) has transformed organizational landscapes, improving operational efficiency, data analysis, and decision-making processes (Haefner et al., 2021; McElheran et al., 2024). AI-driven technologies, including machine learning algorithms, natural language processing, and intelligent chatbots, enhance communication, collaboration, and information dissemination—key elements of organizational success, innovation, and competitive advantage (Jarrahi, 2018; Wang and Noe, 2010). As organizations increasingly rely on AI to automate routine tasks, employees can focus on more complex problem-solving, fostering continuous learning and innovation, which is essential for sustained competitiveness (Argote and Miron-Spektor, 2011; Haefner et al., 2021).

AI tools also facilitate the efficient distribution of knowledge throughout organizations by providing real-time access to information, which enables employees to collaborate from various locations (Haefner et al., 2021; Huang and Rust, 2018). These technologies help identify knowledge gaps and customize learning pathways, promoting continuous professional development and enhancing knowledge transfer (Dwivedi et al., 2021; Mikalef and Gupta, 2021). Additionally, AI’s role in knowledge sharing can be amplified by providing specialized training and continuous development opportunities. This enhances employees’ proficiency in utilizing AI tools, fostering engagement in knowledge-sharing activities, and contributing to a collaborative organizational culture (Jarrahi, 2018; Mikalef and Gupta, 2021).

The adoption of AI has significant implications for enhancing employee knowledge sharing (Argote and Miron-Spektor, 2011; Haefner et al., 2021). This is because the adoption of AI brings many new opportunities. On the one hand, it can accelerate employees’ exposure to new knowledge, and on the other hand, the collaboration between AI and employees provides new ways of working (Jarrahi, 2018). Although AI presents substantial benefits, its effective integration into knowledge sharing entails considerable challenges. What scholars do not know yet is the role that leadership style and employees’ attitudes toward new technologies play in this process. Specifically, the success of AI adoption for knowledge sharing is significantly influenced by employees’ perceptions of technology and leadership styles. Employees exhibiting high technophilia are more inclined to adopt AI, thereby improving learning and collaboration (Charness and Boot, 2009; Ronit, 2011). Conversely, individuals with diminished technophilia may oppose integration, constraining its potential. In addition, employees who work with paradoxical leaders are more likely to view AI adoption as an opportunity for professional and personal development, exhibiting more positive emotions and motivation to learn. Technophilia and paradoxical leadership may play significant conditional roles in the correlation between AI adoption and efficient knowledge sharing (Venkatesh et al., 2012).

**Figure 1:**
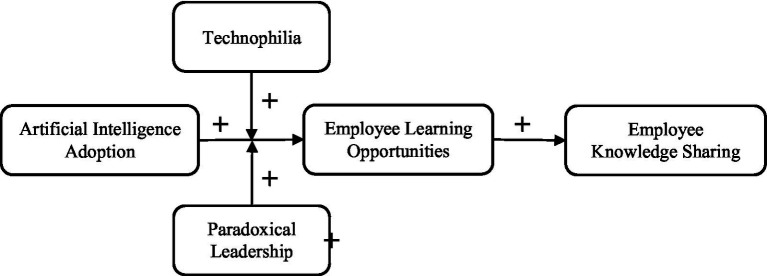
Theoretical model.

This study aims to investigate the mediating role of learning opportunities in the correlation between AI adoption and knowledge sharing while also analyzing the moderating effects of paradoxical leadership and technophilia on this dynamic. Although extensive research has examined the operational advantages of AI, few studies have explored how learning opportunities can facilitate the connection between AI adoption and employee knowledge sharing, as well as the impacts of leadership style and individuals’ technological attitudes on this relationship (Haefner et al., 2021; McElheran et al., 2024). This research, drawing on social cognition theory, examines how organizations can improve employees’ capacity to utilize AI by aligning learning opportunities with different degrees of technophilia and paradoxical leadership. The study aims to provide practical insights through the development of a mediation-moderation model, focusing on how nurturing a supportive learning leadership and comprehending technological attitudes can enhance AI’s contribution to employee knowledge sharing (Bandura, 1977; Dwivedi et al., 2021). See Figure 1 for the theoretical model.

## 2 Literature review and research hypothesis

### 2.1 Social cognition theory

Albert Bandura’s social cognitive theory (SCT) emphasizes the complex interaction among individual factors, behaviors, and the environment in influencing human actions. The theory fundamentally presents the notion of reciprocal determinism, which posits that cognition, behaviors, and environmental factors perpetually interact, with each component influencing and being influenced by the others (Ajzen, 1991; Bandura and Cervone, 1983). SCT highlights observational learning, wherein individuals learn new behaviors and skills through the observation of others, and self-regulation, which allows individuals to manage their actions and emotions to attain personal objectives (Bandura, 1991). These principles provide a significant framework for comprehending individual interactions with technology, especially regarding technology adoption, as they emphasize the impact of individual factors, contextual elements, and behavioral responses on the adoption process (Bandura and Cervone, 1983; Davis, 1989; Venkatesh et al., 2003).

Based on this theory, we propose that AI adoption, as an important organizational environmental factor, brings many new opportunities that can drive employees to learn. These learning opportunities in turn drive employees to share their knowledge with others within the organization, resulting in knowledge sharing behavior. Paradoxical leadership and technophilia are important leadership style factors and individual factors that affect this process, respectively. Paradoxical leadership provides flexible and versatile leadership styles, helping employees focus more on self-development (Zhang et al., 2015, 2022), while technophilia encourages individuals to embrace novel technologies and focus on learning new skills and knowledge (Hannan et al., 2023; Seebauer et al., 2015). These two factors serve as critical boundary conditions that drive employees to seek out learning opportunities and engage in knowledge sharing.

### 2.2 AI adoption, employee learning opportunities, and employee knowledge sharing

Organizations are utilizing advanced AI technologies, including machine learning algorithms, natural language processing, and predictive analytics, to provide personalized and adaptive learning experiences (Dong and McIntyre, 2014). These tools enable organizations to customize learning and development opportunities to address the unique needs of individual employees, ensuring alignment with personal growth and organizational objectives (Jarrahi, 2018). This shift bridges the gap between conventional learning approaches and the rapidly changing requirements of the contemporary workforce, enhancing the efficiency and specificity of learning (Choudhary et al., 2023). AI-driven platforms can assess employees’ learning preferences and adapt content delivery, establishing customized learning paths that maximize engagement, knowledge retention, and comprehensive skill advancement (Qazi et al., 2023).

Beyond improving individual learning, the adoption of AI is crucial for automating repetitive tasks, thus freeing up cognitive resources for employees to concentrate on more intricate and innovative learning endeavors (Glikson and Woolley, 2020). Artificial intelligence tools, including intelligent assistants and recommendation systems, assist in the acquisition of pertinent information, directing employees to resources that augment their learning experience (Olan et al., 2022). This automation promotes a culture of autonomous learning, enabling employees to recognize their learning gaps and independently pursue solutions, thereby improving job satisfaction and motivation (Bodea et al., 2024; Orces et al., 2005). Consequently, AI goes beyond being a simple tool for learning; it becomes an integral partner in fostering both personal and professional growth, helping employees to evolve and thrive within their roles, driving them to actively acquire learning opportunities (Huang and Rust, 2018). Thus, we propose:

*Hypothesis 1*: Artificial intelligence adoption is positively related to employee learning opportunities.

Employee learning opportunities are the resources and environments provided by organizations that enable employees to acquire new skills and knowledge (Bowyer and Kahne, 2020). These opportunities are essential for enhancing individual capabilities and fostering continuous improvement culture. In contrast, employee knowledge sharing involves the exchange of information, insights, and expertise among employees to jointly advance organizational goals (Ouakouak et al., 2021). Based on the premise that AI adoption improves learning opportunities, it is essential to explore how these opportunities affect knowledge-sharing behaviors within organizations (Jarrahi, 2018). AI-driven learning systems enhance individual competencies and promote a culture of collaboration and knowledge-sharing (Huang and Rust, 2018; Wang, 2023). AI systems foster personalized and adaptive learning experiences, prompting employees to share their acquired knowledge, thereby establishing a cycle of continuous learning and collective intelligence that improves knowledge sharing (Orces et al., 2005; Ouakouak et al., 2021).

Many researches have shown that individual learning substantially influences employees’ willingness to share knowledge, as they are more inclined to share insights acquired from AI-driven learning tools (Venkatesh and Bala, 2008). AI systems dismantle conventional knowledge silos, enabling unified knowledge transfer and fostering a collaborative atmosphere that enhances both innovation and growth (Huang and Rust, 2018; Wang and Noe, 2010). Moreover, AI augments social connectivity within organizations by facilitating peer-to-peer learning and strengthening a culture of collective learning (Ouakouak et al., 2021).

The notion of psychological empowerment is intricately linked to AI-driven learning systems. As employees acquire new skills via AI-enabled tools, their perception of competence and value within the organization is enhanced, increasing their propensity to share knowledge with others (Seo, 2023). AI learning tools provide real-time feedback and interactive features that enhance employees’ confidence, encouraging their participation in the organization’s knowledge-sharing ecosystem (Seo, 2023; Wang and Noe, 2010).

In the context of AI adoption, structured learning opportunities are increasingly essential. AI-driven learning systems provide employees with the vital tools and confidence to participate in observational and experiential learning, which are crucial for knowledge exchange (Cohen and Levinthal, 1990). AI training, which imparts employees with the competencies to utilize new technologies, has been shown to significantly increase their participation in knowledge-sharing endeavors (Jarrahi, 2018; Mirbabaie et al., 2022).

Immersive learning environments that promote practical engagement with AI technologies are crucial for cultivating employees’ cognitive strategies for collaboration (Mike, 2023). These environments, which prioritize real-time, interactive learning, encourage the adoption of AI tools among employees while cultivating a culture of collaboration and knowledge exchange. Mirbabaie et al. (2022) found that employees engaged in continuous learning via AI-specific training programs are more inclined to share knowledge, especially through sophisticated technological platforms (Mirbabaie et al., 2022). This dynamic establishes a feedback loop in which knowledge sharing is pivotal to the adoption and utilization of AI tools within the organization.

*Hypothesis 2*: Employee learning opportunities are positively related to employee knowledge sharing.

*Hypothesis 3*: Artificial intelligence adoption is positively and indirectly related to employee knowledge sharing via employee learning opportunities.

### 2.3 The moderating role of paradoxical leadership

Paradoxical leadership is a modern style that involves a range of leader u that may seem initially contradictory but are fundamentally interrelated (Zhang et al., 2015; Zhang and Liu, 2022). These behaviors effectively address and balance the competing demands of the workplace over the long term. This form of leadership consists of five dimensions: (1) combining self-centeredness with other-centeredness; (2) maintaining both distance and closeness; (3) treating subordinates uniformly while allowing individualization; (4) enforcing work requirements while allowing flexibility; (5) maintaining decision control while allowing autonomy (Zhang et al., 2015).

According to social cognitive theory, individual behavior is influenced by environmental cues (Bandura, 1991), and leadership style is one of the key cues that employees use to perceive the work environment. In the context of AI adoption, paradoxical leaders communicate positive signals to employees by creating conjoint bounded and discretionary work environments (Zhang et al., 2015), which are characterized by engagement, transparency, and inclusiveness. This style of leadership meets the basic needs of employees, stimulates their motivation for autonomy, and prompts positive thinking (Lin et al., 2024). As a result, when faced with AI adoption, employees are more likely to view the widespread use of AI as an opportunity for professional and personal development rather than a threat (Bandura, 1991), thereby prompting them to be more active in acquiring learning opportunities (LePine et al., 2016). In contrast, employees who lack paradoxical leadership often struggle to achieve balance when faced with opposing needs and goals. Their lack of information and motivation in responding to AI adoption makes them more likely to perceive AI as an obstacle that poses a potential threat to their personal growth or interests (Cheng et al., 2023), resulting in resistance to AI technology, which in turn affects their utilization of learning opportunities. Based on this, we propose the following hypothesis:

*Hypothesis 4*: Paradoxical leadership moderates the positive relationship between artificial intelligence adoption and employee learning opportunities, such that this relationship is stronger when paradoxical leadership is higher (vs. lower).

Taken together, we propose that paradoxical leadership moderates the indirect effects of AI adoption on employee learning opportunities and knowledge-sharing behaviors. Social cognitive theory states that self-regulation are key mechanisms for shaping behavior (Bandura, 1991). Paradoxical leaders foster initiative by modeling or creating work environments that have both constraints and discretion (Zhang et al., 2015), which motivates employees to explore new strategies to achieve their goals. AI adoption increases learning opportunities and provides timely, high-quality feedback, both of which motivates employees to take advantage of these opportunities through continuous learning (Parker et al., 2021), and to share their insights and experiences with others, thereby creating a cycle of continuous learning and collective wisdom. Specifically, when the paradoxical leadership style is more prominent, employees are more likely to view AI adoption as an opportunity to actively seek out more learning opportunities, which in turn promotes knowledge-sharing behaviors. This is because the positive atmosphere created by paradoxical leadership enables employees to face the changes brought about by AI with an open and proactive mindset, viewing it as a beneficial tool for improving both self and organizational performance. Conversely, employees with lower levels of paradoxical leadership are more likely to perceive AI adoption as an obstacle due to their difficulty in balancing the complex and contradictory needs and challenges in the organization, thus reacting passively to the current situation. They may behave in ways that hinder their learning behaviors, resist learning opportunities, and have difficulty adapting to new technologies, and this resistance can further inhibit their knowledge exchange within the organization.

*Hypothesis 5*: Paradoxical leadership moderates the positive indirect effect of artificial intelligence adoption on employee knowledge sharing via employee learning opportunities, such that this indirect effect is stronger when paradoxical leadership is higher (vs. lower).

### 2.4 The moderating role of technophilia

Employees signifying significant technophilia often view new technologies not just as instruments for increasing productivity but as opportunities for personal and professional development (Venkatesh et al., 2003). They perceive AI as a conduit for acquiring new skills, enhancing their knowledge, and augmenting job performance (Agrawal et al., 2019; Ronit, 2011; Venkatesh et al., 2012). This favorable attitude toward technology promotes increased engagement with AI, facilitating ongoing skill development, innovation, and enhanced job satisfaction (Saraih et al., 2024).

Individuals with a high degree of technophilia view technology as a tool to fuel growth rather than as a threat. According to social cognitive theory, individual factors influence their cognitive and behavioral responses (Bandura, 1977). Such employees are more likely to learn effectively by observing the behavior of colleagues or leaders who have successfully integrated AI into their workflow (Gable et al., 2008; Saraih et al., 2024). They actively seek out and adopt AI technologies, recognizing them as powerful tools for enhancing individual performance and achieving organizational goals. Their intrinsic motivation to explore new technologies enhances their confidence in mastering AI tools and motivates them to actively try out AI systems, thereby accelerating the AI adoption process (Venkatesh and Bala, 2008). This perception is consistent with the emphasis on outcome expectations as a driver of behavioral intentions in social cognitive theory. As a result, highly technophilic employees are more likely to view AI adoption positively, as an opportunity for personal and professional growth rather than as a threat (Seebauer et al., 2015). This positive attitude may promote employees’ use of AI tools, which in turn increases their learning opportunities. Conversely, employees with lower levels of technophilia may lack the curiosity or confidence to engage deeply with AI applications, or even view them as a disruptive force rather than an opportunity (Fichman, 2004), which hinders their access to learning opportunities.

*Hypothesis 6*: Technophilia moderates the positive relationship between artificial intelligence adoption and employee learning opportunities, such that this relationship is stronger when technophilia is higher (vs. lower).

Technophilic employees have a positive attitude toward new technologies, and this attitude enhances their intrinsic motivation to use them. As a result, they are more likely to integrate AI-driven insights into their workflows and exhibit higher levels of engagement with technology (Gable et al., 2008; Saraih et al., 2024). They view AI as a means to enhance their skills and job performance (Venkatesh and Bala, 2008), and this intrinsic motivation drives them to take advantage of learning opportunities, ultimately leading to frequent knowledge-sharing activities. Social cognitive theory emphasizes the importance of observational learning, in which employees learn by observing others (Bandura, 1977). Technophilic employees are more likely to adopt AI-driven learning practices and set an example for their co-workers, becoming role models for other employees. This creates a ripple effect that promotes knowledge sharing throughout the organization. Conversely, employees with lower levels of technophilia may have difficulty relating AI-driven insights to real-world situations, which limits their willingness or ability to share knowledge effectively (Fichman, 2004). For these employees, AI adoption is frequently perceived as an external challenge rather than an opportunity, thereby constraining the potential advantages of technological progress. This perception hinders their access to learning opportunities and ultimately impedes knowledge sharing.

*Hypothesis 7*: Technophilia moderates the positive indirect effect of artificial intelligence adoption on employee knowledge sharing via employee learning opportunities, such that this indirect effect is stronger when Technophilia is higher (vs. lower).

## 3 Research method

### 3.1 Sample and procedure

We collaborated with a large Chinese travel company on this study, as the travel industry extensively uses AI in customer service and data analytics to improve operational efficiency and customer experience, which provides a rich context for studying the workplace impact of AI. In this research, data were gathered from 364 employees via a three-wave approach. The participants were all employed by a major Chinese travel company. Access to the organization was granted through contact with its executives by one of the authors. Initially, 460 employees were randomly chosen in collaboration with the company’s HR department to form the research pool. A circular was then disseminated to all selected employees by the HR department, highlighting the senior management’s support for the study and clarifying the survey’s purpose, limitations, anonymity, and voluntariness. Each participant signed an informed consent form. In order to avoid common methodological biases, the time interval between each wave was 2 weeks (Podsakoff et al., 2003). Specifically, at Time 1, employees were asked to rate artificial intelligence adoption paradoxical leadership, technophilia age, gender, education, and tenure. At Time 2, employees were invited to report employee learning opportunities. At Time 3, employees responded to employee knowledge sharing.

After sorting and culling the collected data, we collected 364 valid responses (with a final response rate of 79.13%). The average age of employees was 31.000 years (*SD* = 5.284), with an average tenure of 5.165 years (*SD* = 3.053). Among them, 77.198% held a bachelor’s degree or higher, and 57.142% were male.

### 3.2 Measures

In this study, all items were presented in Chinese, and we strictly followed Brislin’s (1986) standard translation and back-translation procedures (Brislin, 1986). Response options ranged from 1 (*strongly disagree*) to 7 (*strongly agree*).

Consistent with the study by Tang et al. (2022), artificial intelligence adoption was measured using Medcof’s (1996) three items (Medcof, 1996). A sample item is “I used artificial intelligence to carry out most of my job functions.” We measure paradoxical leadership using Zhang et al.’s (2015) 22-item scale (Zhang et al., 2015). A sample item is “Uses a fair approach to treat all subordinates uniformly, but also treats them as individuals.” Technophilia was rated by employees using Wolff and Madlener’s (2019) four-item scale (Wolff and Madlener, 2019). A sample item is “I am always interested in using the newest technical devices.” We adapted Bowyer and Kahne’s (2020) three items to assess employee learning opportunities (Bowyer and Kahne, 2020). A sample item is “In this organization, I have opportunities to learn about societal issues that I care about.” We used Ouakouak et al.’s (2021) five-item scale to capture employee knowledge seeking (Ouakouak et al., 2021). A sample item is “I usually exchange information knowledge and share skills with my coworkers.”

Consistent with previous literature on AI research (Luo et al., 2021; Yam et al., 2021), we control for employees’ age, gender, education, and tenure.

## 4 Results

In this paper, the analyses were performed using Mplus 8.3 and SPSS 26.0. Confirmatory factor analysis (CFA) and structural equation modeling (SEM) were conducted via Mplus 8.3. Descriptive statistics and correlation analyses were performed using SPSS 26.0. Additionally, the PROCESS macro was employed to test the moderating and mediating effects using a bootstrap method with 10,000 resamples. The means, standard deviations, reliabilities, and correlations of study variables is presents in Table 1. The Harman’s single-factor test showed that the first factor loading was 36.767% (< 40%) indicating that the common method bias was not a serious threat in this study (Podsakoff et al., 2003). Additionally, Cronbach’s alphas of key variables ranged from 0.898 to 0.977, composite reliabilities (CR) ranged from 0.900 to 0.978, and average variances extracted (AVE) ranged from 0.614 to 0.936 (see Table 2). As shown in Table 3, the hypothesized five-factor model (*χ^2^* = 1476.958, *df* = 619, CFI = 0.93, TLI = 0.93, RMSEA = 0.06, SRMR = 0.04) fits the data better than other alternative measurement models.

**Table 1:** Descriptive statistics and correlations among study variables.

Variables	1	2	3	4	5	6	7	8	9
1. Age	–								
2. Tenure	0.764^**^	–							
3. Gender	−0.045	−0.035	–						
4. Education	−0.051	−0.059	0.101	–					
5. Artificial intelligence adoption	0.028	−0.053	0.009	−0.032	**0.968**				
6. Paradoxical leadership	0.024	−0.022	−0.014	0.020	−0.089	**0.784**			
7. Technophilia	0.068	0.068	0.003	0.092	−0.035	0.065	**0.924**		
8. Employee learning opportunities	0.101	0.037	0.002	0.007	0.373^**^	0.129^*^	−0.038	**0.866**	
9. Employee knowledge sharing	−0.024	−0.073	0.009	0.054	0.369^**^	0.079	−0.051	0.412^**^	**0.846**
Mean	31.000	5.165	0.571	3.055	4.087	4.105	4.519	4.032	4.009
SD	5.284	3.053	0.496	0.910	1.909	1.163	1.078	0.872	0.746

*n* = 364. ^*^p < 0.05 and ^**^p < 0.01. Gender: 0 = female, 1 = male; Education: 1 = high school diploma or below, 2 = associate’s degree, 3 = bachelor’s degree, 4 = master’s degree or above. Tenure (team tenure) is in years. CR, composite reliability; AVE, average variance extracted. Diagonal elements (in bold) are the square root of the average variance extracted (AVE).

**Table 2:** Results of confirmatory factor analysis.

Variables	Loading	CR	AVE	Cronbach’s α
1. Artificial intelligence adoption	0.958—0.977	0.978	0.936	0.977
2. Paradoxical leadership	0.717—0.863	0.972	0.614	0.972
3. Technophilia	0.906—0.940	0.959	0.853	0.958
4. Employee learning opportunities	0.854—0.878	0.900	0.750	0.898
5. Employee knowledge sharing	0.795—0.897	0.926	0.715	0.925

CR, composite reliability; AVE, average variance extracted.

**Table 3:** Results of confirmatory factor analysis.

CFA models	*χ^2^*	*df*	CFI	TLI	RMSEA	SRMR
Five-factor model (AIA, PL, TC, ELO, EKS)	1476.958	619	0.932	0.927	0.062	0.036
Four-factor model (AIA, PL + TC, ELO, EKS)	7795.409	623	0.433	0.394	0.178	0.338
Four-factor model (AIA, PL, TC, ELO + EKS)	2053.739	623	0.887	0.879	0.079	0.054
Four-factor model (AIA + PL, TC, ELO, EKS)	3193.153	623	0.797	0.783	0.106	0.091
Four-factor model (AIA + TC, PL, ELO, EKS)	3129.809	623	0.802	0.788	0.105	0.089
Three-factor model (AIA, PL + TC, ELO + EKS)	8371.740	626	0.388	0.349	0.184	0.340
Two-factor model (AIA + PL + TC, ELO + EKS)	5392.797	628	0.624	0.601	0.144	0.125
Two-factor model (AIA, PL + TC + ELO + EKS)	9969.101	628	0.262	0.218	0.202	0.359
One-factor model (AIA + PL + TC + ELO + EKS)	6944.786	629	0.501	0.472	0.166	0.160

AIA, Artificial intelligence adoption; PL, Paradoxical leadership; TC, Technophilia; ELO, Employee learning opportunities; EKS, Employee knowledge sharing.

We conducted conditional process analysis to test our hypotheses by using the PROCESS macro based on Preacher et al.’s (2007) recommendations (Hayes, 2022). Table 4 presents the results of regression estimates. In line with our assumption, the relationship between artificial intelligence adoption and employee learning opportunities was significant and positive (*β* = 0.169, *SE* = 0.021, *p* < 0.001). Additionally, the relationship between employee learning opportunities and employee knowledge sharing was significant and positive (*β* = 0.276, *SE* = 0.043, *p* < 0.001). Hence, Hypotheses 1 and 2 were supported.

**Table 4:** Regression results for direct effects and moderation effects.

Variables	Employee learning opportunities			Employee knowledge sharing
	M1	M2	M3	M4
Constant	3.638 (0.335)^***^	3.555 (0.346)^***^	3.749 (0.329)^***^	2.912 (0.321)^***^
Age	0.012 (0.012)	0.015 (0.012)	0.009 (0.012)	−0.003 (0.010)
Tenure	−0.007 (0.021)	−0.002 (0.021)	−0.001 (0.020)	−0.013 (0.018)
Gender	−0.002 (0.082)	0.009 (0.085)	0.005 (0.080)	−0.003 (0.070)
Education	0.020 (0.045)	0.012 (0.046)	0.012 (0.044)	0.045 (0.038)
Artificial intelligence adoption	0.162 (0.021)^***^	0.176 (0.022)^***^	0.169 (0.021)^***^	0.097 (0.020)^***^
Paradoxical leadership	−0.011 (0.035)		−0.018 (0.034)	
Artificial intelligence adoption **×** Paradoxical leadership	0.119 (0.018)^***^		0.117 (0.018)^***^	
Technophilia		0.132 (0.039)^***^	0.133 (0.037)^***^	
Artificial intelligence adoption **×** Technophilia		0.045 (0.020)^*^	0.037 (0.019)^*^	
Employee learning opportunities				0.276 (0.043)^***^
*R^2^*	0.243	0.186	0.277	0.232
*F*	16.321^***^	11.622^***^	15.059^***^	17.923^***^

*n* = 364. ^*^p < 0.05, ^**^p < 0.01, and ^***^p < 0.001. Unstandardized coefficients are reported. Values in parentheses are standard error estimates.

To assess the mediation, moderation, and moderated mediation effects, we conducted conditional process analyses with 10,000 bootstrapped samples to generate 95% confidence intervals for the estimates. Our analysis displayed that artificial intelligence adoption is positively and indirectly related to employee knowledge sharing via employee learning opportunities (indirect effect = 0.047, *SE* = 0.009, 95% CI = [0.030, 0.066]). Thus, this finding supported Hypothesis 3.

**Figure 2:**
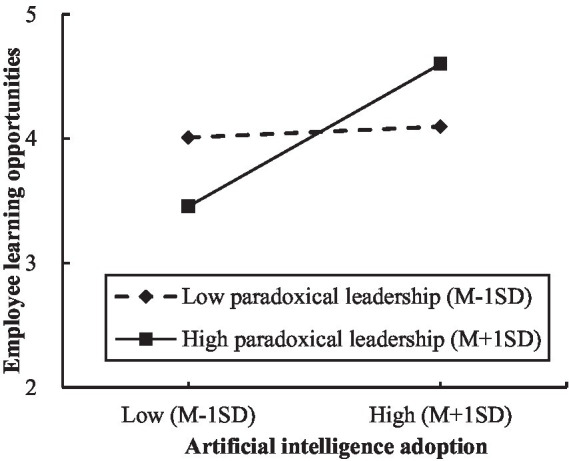
The moderating effect of paradoxical leadership on the relationship between artificial intelligence adoption and employee learning opportunities.

Notably, the data were mean-centered prior to testing our moderated and moderated mediation hypotheses (Aiken and West, 1991). Table 4 demonstrated that the interaction effect between artificial intelligence adoption and paradoxical leadership was significant and positive (*β* = 0.119, *SE* = 0.018, *p* < 0.001). Furthermore, we plotted the relationship between artificial intelligence adoption and employee learning opportunities at high (Mean + 1SD) and low (Mean − 1SD) levels of paradoxical leadership (Aiken and West, 1991). The Figure 2 revealed that the relationship between artificial intelligence adoption and employee learning opportunities was significant and stronger when paradoxical leadership was higher (*β* = 0.300, *SE* = 0.029, 95% CI = [0.243, 0.357]), whereas it was nonsignificant when paradoxical leadership was lower (*β* = 0.023, *SE* = 0.031, 95% CI = [−0.037, 0.083]). Thus, Hypothesis 4 was supported.

Furthermore, the conditional process analysis revealed that the positive indirect effect of artificial intelligence adoption on employee knowledge sharing through employee learning opportunities was significantly stronger when paradoxical leadership was higher (*β* = 0.083, *SE* = 0.015, 95% CI = [0.055, 0.114]), compared with when paradoxical leadership was lower (*β* = 0.006, *SE* = 0.009, 95% CI = [−0.012, 0.024]). Moreover, the contrast of these two conditional indirect effects was significant (*Δ IND* = 0.077, *SE* = 0.018, 95% CI = [0.045, 0.113]). Hence, the results supported Hypothesis 5.

**Figure 3:**
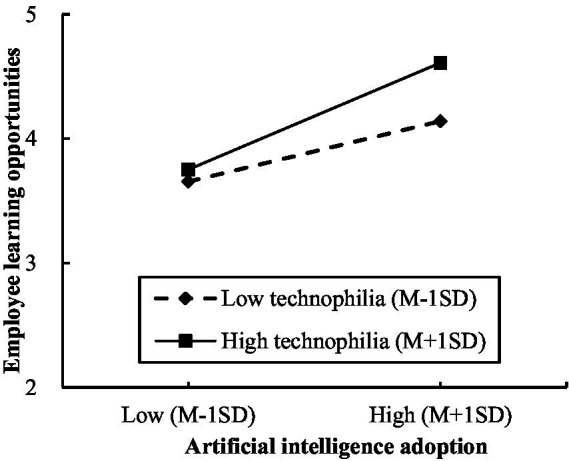
The moderating effect of technophilia on the relationship between artificial intelligence adoption and employee learning opportunities.

Similarly, Table 4 demonstrated that the interaction effect between artificial intelligence adoption and technophilia was significant and positive (*β* = 0.045, *SE* = 0.020, *p* < 0.05). Furthermore, we plotted the relationship between artificial intelligence adoption and employee learning opportunities at high (Mean + 1SD) and low (Mean −1SD) levels of technophilia (Aiken and West, 1991). The Figure 3 revealed that the relationship between artificial intelligence adoption and employee learning opportunities was significant and stronger when technophilia was higher (*β* = 0.225, *SE* = 0.031, 95% CI = [0.165, 0.285]), whereas it was significant and weaker when technophilia was lower (*β* = 0.127, *SE* = 0.031, 95% CI = [0.067, 0.187]). Thus, Hypothesis 6 was supported.

Furthermore, the conditional process analysis revealed that the positive indirect effect of artificial intelligence adoption on employee knowledge sharing through employee learning opportunities was significantly stronger when technophilia was higher (*β* = 0.062, *SE* = 0.013, 95% CI = [0.039, 0.088]), compared with when technophilia was lower (*β* = 0.035, *SE* = 0.010, 95% CI = [0.018, 0.056]). Moreover, the contrast of these two conditional indirect effects was significant (*Δ IND* = 0.027, *SE* = 0.012, 95% CI = [0.005, 0.052]). Hence, the results supported Hypothesis 7.

## 5 Discussion

### 5.1 Theoretical implications

This study provides substantial contributions to the existing literature on AI adoption, employee knowledge sharing, paradoxical leadership, technophilia, and employee learning opportunities. First, this study clarified the significance of AI adoption as a critical facilitator of employee learning opportunities and knowledge-sharing behaviors. Earlier studies assumed a direct link between AI and knowledge sharing (Olan et al., 2022), but failed to explain how learning opportunities translate into sharing behaviors. This research employed social cognition theory (Bandura, 1977) to demonstrate how employees observe and engage with AI tools to acquire new skills and competencies. The adoption of AI transcended mere technical implementation, cultivating a learning-centric environment by equipping employees with tools that promote knowledge sharing. This framework builds upon previous research regarding AI’s capacity to enhance collaboration by highlighting its influence on employees’ cognitive and behavioral patterns (Jarrahi, 2018; Mirbabaie et al., 2022).

Second, the research identified paradoxical leadership and technophilia as moderating variables in the relationship between AI adoption and employee learning opportunities, thus offering a distinctive contribution to the understanding of leadership style and individual attitudes toward technology. Our research clarifies for the first time the positive effects of paradoxical leadership on AI adoption, emphasizing that leaders can foster employee initiative by creating conjoint bounded and discretionary work environments, thereby encouraging employees to engage in learning activities and knowledge exchange. These findings fill the research gap on leadership styles in AI-related workplaces (Zhang et al., 2015; Lin et al., 2024), expanding our understanding of AI and leadership styles. In addition, technophilia, characterized by an intense enthusiasm for technology, was found to enhance the likelihood of employees employing AI tools, thereby facilitating their learning and development. In contrast, employees with low technophilia may struggle to adapt to AI implementation, thereby limiting their engagement in learning opportunities. This finding underscores the need for organizations to tailor AI adoption and training initiatives to leadership style and individual technophilic tendencies, ensuring more effective engagement and smoother integration of AI technologies in the workplace (Venkatesh and Bala, 2008).

Third, the study highlighted the importance of employee learning opportunities as a vital mediating factor between AI adoption and employee knowledge sharing. The study emphasized that the adoption of AI enhances learning pathways, suggesting that employee engagement with AI tools promotes skill acquisition and enhances their capacity to share knowledge with others (Argote and Miron-Spektor, 2011). This process aligns with the principles of social cognition theory, which underscores the importance of observed behaviors and interactions in shaping cognitive and learning results (Bandura, 1991). In this context, employee learning opportunities serve as a mechanism that transforms AI adoption into actionable knowledge-sharing practices, promoting a more collaborative and innovative work environment. By combining these perspectives, our study offers a comprehensive framework to explain how technology and human thinking interact and develop together in knowledge-focused settings, this improves our understanding of how organizational investments in AI-driven learning environments can directly facilitate knowledge exchange among employees (Alerasoul et al., 2022).

### 5.2 Practical implications

This study provides essential guidance for organizations aiming to improve knowledge-sharing practices and leverage the transformative potential of AI. Successful AI adoption requires tackling both technological and organizational obstacles while fostering an environment that promotes effortless knowledge sharing. Organizations can empower employees with the necessary proficiency to effectively utilize AI tools by implementing tailored training initiatives and skill-building programs. Teams can also invest in AI-powered learning platforms that provide personalized and adaptive learning experiences. These platforms should be designed to meet the unique needs of individual employees, ensuring alignment with personal growth and organizational goals. These efforts promote innovation, collaboration, and peer engagement, ultimately leading to significant improvements in overall performance (Deng et al., 2023).

Moreover, customizing AI adoption strategies based on leadership styles (e.g., paradoxical leadership) and individual differences (e.g., technophilia) is essential for enhancing adoption outcomes. Our findings suggest that paradoxical leadership and technophilia amplifies the impact of AI on learning, and employees inclined toward adopting technology and paradoxical leadership styles are more likely to successfully incorporate AI systems into their workflows (Davis, 1989; Venkatesh et al., 2003). Tailored training programs that are aligned with employees’ technological readiness can promote a seamless transition and improve adoption rates, ensuring the complete integration of AI technologies into organizational processes. For example, AI training programs can be tailored to employees’ technophilic tendencies, ensuring that employees with lower levels of technophilia receive additional support to overcome resistance to using AI tools and build confidence.

### 5.3 Limitations and future research directions

This study provides significant insights into the effects of AI adoption on knowledge sharing; however, certain limitations should be recognized. First, although this study controls for variables such as employee tenure, it does not delve into their dynamic relationships with AI adoption and knowledge sharing. Indeed, while the new generation of employees is more receptive to new technologies, senior employees tend to exhibit higher levels of commitment—characteristics that can be leveraged to drive AI adoption and knowledge sharing. Therefore, future research could examine the impact of employee tenure and commitment on AI adoption and knowledge sharing to better understand the complex mechanisms involved. Second, while social cognitive theory provides a powerful framework for understanding how employees interact with AI, it may not fully capture the complexity of AI adoption in different organizational contexts. Therefore, future research could explore additional theoretical perspectives, such as the Technology Acceptance Model (TAM) or the Unified Theory of Acceptance and Use of Technology (UTAUT), to complement social cognitive theory and provide a more comprehensive understanding of AI adoption and its outcomes. Third, the sample for this study was drawn from a single organization in the tourism industry, which may limit the generalizability of the findings. Future research could expand the sample to include multiple organizations in different industries (e.g., manufacturing vs. services) to enhance the external validity of the findings. Additionally, although we adopted a time-lag research method, the perspective of this study was static, and future research can consider a longitudinal dynamic perspective and corresponding research methods.

## Data Availability

The raw data supporting the conclusions of this article will be made available by the authors, without undue reservation.
