# Supplementary material for: How and when artificial intelligence adoption promotes employee knowledge sharing? The role of paradoxical leadership and technophilia
Source: Front Psychol. 2025 May 21;16:1573587. doi: 10.3389/fpsyg.2025.1573587 (PMC12134625; doi:10.3389/fpsyg.2025.1573587)
Supplement: Supplementary file 1 [file Table_1.docx]

**Appendix-Scales**

**Artificial intelligence adoption**

1. I used artificial intelligence to carry out most ofmy job functions.

2. Ispent most of the time working with artificial intelligence.

3. I worked with artificial intelligence in making major work decisions.

**Paradoxical leadership**

1. Uses a fair approach to treat all subordinates uniformly, but also treats them as individuals.

2. Puts all subordinates on an equal footing, but considers their individual traits or personalities.

3. Communicates with subordinates uniformly without discrimination, but varies his or her communication styles depending on their individual characteristics or needs.

4. Manages subordinates uniformly, but considers their individualized needs.

5. Assigns equal workloads, but considers individual strengths and capabilities to handle different tasks.

6. Shows a desire to lead, but allows others to share the leadership role.

7. Likes to be the center of attention, but allows others to share the spotlight as well.

8. Insists on getting respect, but also shows respect toward others.

9. Has a high self-opinion, but shows awareness of personal imperfection and the value of other people.

10. Is confident regarding personal ideas and beliefs, but acknowledges that he or she can learn from others.

11. Controls important work issues, but allows subordinates to handle details.

12. Makes final decisions for subordinates, but allows subordinates to control specific work processes.

13. Makes decisions about big issues, but delegates lesser issues to subordinates.

14. Maintains overall control, but gives subordinates appropriate autonomy.

15. Stresses conformity in task performance, but allows for exceptions.

16. Clarifies work requirements, but does not micro- manage work.

17. Is highly demanding regarding work performance, but is not hypercritical.

18. Has high requirements, but allows subordinates to make mistakes.

19. Recognizes the distinction between supervisors and subordinates, but does not act superior in the leadership role.

20. Keeps distance from subordinates, but does not remain aloof.

21. Maintains position differences, but upholds subordinates’ dignity.

22. Maintains distance from subordinates at work, but is also amiable toward them.

**Technophilia**

1. I usually like to install interesting new apps.

2. I regularly use apps for payments, reservations, errands, etc.

3. I am enthusiastic about GPS and travel apps.

4. I think it is exciting to try new apps.

**Employee learning opportunities**

1. I have discussed current events and/or controversial issues.

2. I have learned about societal issues that I care about.

3. I am encouraged to consider multiple views on controversial issues.

**Employee knowledge sharing:**

1. If I have some special knowledge or technique on how to perform a task, I am likely to tell others about it.

2. I usually exchange information knowledge and share skills with my coworkers.

3. I freely provide other members with hard-to-find knowledge or specialized skills.

4. I voluntarily help others to develop relevant strategies and techniques to perform their work.

5. I share a lot of useful work-related information and ideas with others.
